# Vaccine Hesitancy and Associated Factors Amongst Health Professionals: A Scoping Review of the Published Literature

**DOI:** 10.3390/vaccines12121411

**Published:** 2024-12-13

**Authors:** Antonios Christodoulakis, Izolde Bouloukaki, Antonia Aravantinou-Karlatou, Michail Zografakis-Sfakianakis, Ioanna Tsiligianni

**Affiliations:** 1 Department of Social Medicine, School of Medicine, University of Crete, 71500 Heraklion, Greece; christodoulakisa@uoc.gr (A.C.); medp2012149@med.uoc.gr (A.A.-K.); i.tsiligianni@uoc.gr (I.T.); 2 Department of Nursing, School of Health Sciences, Hellenic Mediterranean University, 71410 Heraklion, Greece; mzografakis@hmu.gr

**Keywords:** vaccination hesitancy, booster dose, healthcare professionals, COVID-19, reasons, factors, review

## Abstract

**Background/Objectives:** Healthcare professionals (HCPs) hold significant influence over public attitudes toward vaccinations. Studies suggest that HCPs are hesitant towards the coronavirus disease 2019 (COVID-19) vaccines. This hesitancy could lead to lower vaccination rates in the community. Therefore, this scoping review aimed to assess the extent of hesitancy towards COVID-19 booster doses among HCPs and identify the associated factors. **Methods:** A comprehensive search was conducted in the PubMed and Scopus databases from April to August 2024, using keywords related to COVID-19, vaccine hesitancy, HCPs, and booster vaccination. Studies that had been peer-reviewed, published in English after 2022, and focused on the hesitancy of the COVID-19 booster dose hesitancy among HCPs were included. Out of the 6703 studies screened, 24 studies were included. **Results:** Most of the HCPs have received their initial series of COVID-19 vaccinations. However, there is a lower rate of uptake for booster doses, with hesitancy rates ranging from 12% to 66.5%. Hesitancy rates varied significantly across continents, with Asia, Africa, and Europe ranging from 19.7% to 66.5%, 27% to 46.1%, 14% to 60.2%, respectively. Hesitancy was reported to be influenced by various factors, including concerns about vaccine safety, necessity, and effectiveness of these vaccines. In addition, the hesitancy regarding booster doses was also found to be influenced by factors like age, gender, profession, and previous COVID-19. Physicians, nurses, and pharmacists exhibited vaccine hesitancy rates ranging from 12.8% to 43.7%, 26% to 37%, and 26% to 34.6%, respectively. **Conclusions:** Our review underscores the hesitancy among HCPs towards receiving booster doses across countries around the world and explores the underlying factors. These findings provide valuable insights for the design of future pandemic vaccination programs.

## 1. Introduction

The outbreak of the coronavirus disease 2019 (COVID-19) has caused a worldwide emergency situation, affecting various aspects of human life [1]. The unprecedented onset of the COVID-19 pandemic has also imposed a significant burden on healthcare systems worldwide [1]. Measures like social distancing, wearing face masks in public, lockdowns, and quarantines have helped to initially control the transmission of the virus [2,3]. However, to return to normal life, long-term solutions such as universal vaccination were needed [2,4,5]. In this context, the development of COVID-19 vaccines has been suggested to combat the pandemic, by reducing the severity of illness and lowering the spread of the virus [6,7]. Nevertheless, even though the pandemic has been effectively controlled, the virus is still spreading, due to the emergence of new strains of COVID-19 and the declining effectiveness of primary doses of the COVID-19 vaccines [8,9,10]. This has prompted the World Health Organization (WHO) to consistently update its recommendations for COVID-19 vaccination. These updates emphasize the importance of vaccinating both the general public and healthcare professionals (HCPs) [11].

HCPs are frequently considered a target population for vaccination initiatives, as they possess characteristics typically associated with vaccine acceptance. These include a high level of education, clinical experience, and affiliation with professional organizations that advocate for vaccination [12]. Moreover, HCPs were prioritized for the COVID-19 vaccine due to their occupational exposure and frequent interaction with infected individuals [13]. However, their willingness to get vaccinated varied and often fell short of expectations [14]. Encouraging positive attitudes towards vaccination among HCPs was proposed not only for ensuring their personal safety, as well as the safety of their families and patients, but also to promote its acceptance among others [15]. The reason behind this is that HCPs act as facilitators and communicators for vaccines to patients and the general public [16,17,18]. More specifically, during both the initial and subsequent COVID-19 vaccination campaigns, HCPs had the role of addressing concerns and misunderstandings about COVID-19 vaccination, and supporting the benefits of getting vaccinated [19,20]. Several studies have reported resistance to receiving COVID-19 booster doses (BDs) which could potentially negatively impact trust in vaccines among the general population [17,21,22]. If HCPs exhibit hesitancy or resistance towards receiving the vaccine, it could reflect a similar attitude among patients, influencing public opinion [23].

The European Centre for Disease Prevention and Control defines vaccine hesitancy as the “delay in acceptance or refusal of vaccines despite availability of vaccination services” [24]. The distribution of initial vaccine hesitancy among HCPs appears to vary, with certain characteristics such as age, sex, race/ethnicity, political affiliation, professional role, and healthcare facility type identified as predictors of vaccine uptake [25,26,27,28,29,30]. Furthermore, the way HCPs perceive their vulnerability to and the seriousness of COVID-19, as well as their past experience testing positive for the virus, greatly impacted their decision to get vaccinated [26,29]. Importantly, HCPs’ initial hesitancy towards vaccinations appears to remain when it comes to receiving BDs of the COVID-19 vaccine, although studies indicate varying levels of acceptance and hesitancy towards BDs [31,32,33,34]. Based on the initial findings, HCPs viewed BDs as less important and expressed a lack of confidence in them [35]. Recent data on vaccine uptake indicates that the significance of perceiving COVID-19 vaccination as necessary seems to diminish, with a lower number of HCPs receiving BDs compared to those who received the initial two doses [36]. Due to the dynamic nature of the virus, the potential for viral mutations, and the likelihood of declining immunity, understanding HCPs’ hesitancy to receive regular COVID-19 vaccines has significant value for guiding vaccination campaigns.

While concerns regarding vaccine hesitancy among HCPs remain, potentially contributing to disparities in BD uptake, few studies have thoroughly examined the differences in characteristics between HCPs who have received COVID-19 BDs and those who have not. Therefore, the aim of this scoping review was to assess the level of COVID-19 vaccine hesitancy among HCPs and identify the underlying factors that contribute to this hesitancy.

## 2. Materials and Methods

This scoping review followed the JBI guidelines for conducting scoping reviews [37], and the findings were reported using the PRISMA-ScR checklist Supplementary Materials Table S1 [38]. The research protocol was retrospectively registered (protocol number: INPLASY2024100036) on the International Platform of Registered Systematic Review and Meta-analysis Protocols (INPLASY) at https://doi.org/10.37766/inplasy2024.10.0036 in October 2024, accessed on 9 October 2024.

### 2.1. Eligibility Criteria

This scoping review focused on two key questions: What is the prevalence of COVID-19 hesitancy among HCPs worldwide, and what are the factors that contribute to this hesitancy? Our scope was global, encompassing studies from all geographical locations without any specific focus on a particular population. This inclusivity ensured that our review captured a diverse range of perspectives and settings. Therefore, in order to be included in this review, studies had to meet the following criteria: (1) being peer-reviewed articles published in English, (2) focusing on HCPs, (3) investigating COVID-19 vaccine hesitancy, acceptance on booster vaccination, and (4) being published from January 2022 onwards. The exclusion criteria included articles that were not peer-reviewed, editorials, opinion pieces, and studies that focused on non-healthcare professional populations. The study selection process included two stages: screening titles and abstracts, and then conducting a full-text review. Two reviewers independently performed the screening process. Differences among reviewers were addressed through discussion and by involving a third reviewer to reach consensus. To maintain transparency and reproducibility, we employed a PRISMA flow diagram to document the selection process.

### 2.2. Information Sources and Search

A comprehensive approach was taken to find relevant literature through a search strategy. To ensure comprehensive coverage of medical and scientific journals, a literature search was performed in two major literature databases, PubMed and Scopus, from April to August 2024. The search terms used were a mix of keywords and Medical Subject Headings (MeSHs) related to COVID-19, vaccine hesitancy, healthcare professionals, and booster vaccinations. Therefore, we employed in both databases the following keyword combinations and Boolean operators (AND, OR): “COVID-19”, “vaccine hesitancy”, “healthcare professionals”, and “booster vaccination”. The search method was based on prior systematic reviews which analyzed vaccine hesitancy towards the COVID-19 vaccine in general populations and HCPs [17,31].

### 2.3. Data Extraction and Analysis

In this scoping review, we analyzed the data regarding regular COVID-19 vaccination hesitancy in HCPs. This entailed the extraction of information about study design, prevalence of COVID-19 vaccination hesitancy, knowledge, attitudes and factors associated with it in HCPs, of full texts and related results of the included studies by two reviewers using a standardized form for data extraction. After removing duplicates, two independent reviewers evaluated each extraction form, and discussed any discrepancies in a thorough appraisal process. In the extracted data, details about the studies, including the author, year, and country, were added. It also provided information about the participants, such as their profession and sample size. The data encompassed the study design, key findings related to vaccine hesitancy and acceptance, any barriers and challenges that were identified, as well as the coping strategies that were utilized. Two independent reviewers performed the data extraction to guarantee accuracy and consistency. All disagreements during the review’s inclusion phase were resolved through discussion to reach a consensus. In instances where reviewer consensus was not achieved, a third, independent reviewer was employed for arbitration. We employed thematic analysis in our research to categorize factors associated with vaccine hesitancy [39]. Thematic analysis included identifying, analyzing, and reporting patterns in the data.

## 3. Results

### 3.1. Screening and Procedure

**Figure 1:**
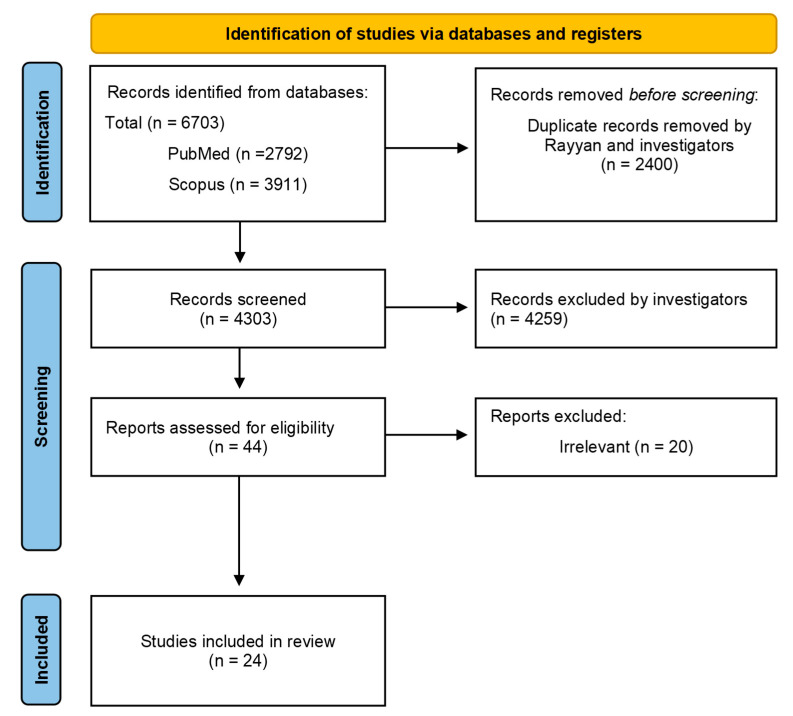
PRISMA 2020 flow diagram for new systematic reviews which included searches of databases and registers only for this scoping review.

A total of 6703 studies were yielded during the first database search for this scoping review. Following the first screening and removal of duplicates, a total of 4303 articles underwent screening based on their titles. Afterwards, 880 titles met the inclusion criteria and were chosen for further assessment, primarily based on their abstracts. Subsequently, a second/further evaluation was conducted on 44 abstracts that satisfied the inclusion criteria, and their full texts were obtained to be further screened. However, 20 studies were excluded in accordance with the criteria for inclusion/exclusion as described in the methodology. Therefore, 24 full-text studies were finally included in this scoping review. The PRISMA flow diagram in Figure 1 shows the process for the literature search.

### 3.2. Overview of Characteristics of the Included Studies

The characteristics of the 24 included studies [22,40,41,42,43,44,45,46,47,48,49,50,51,52,53,54,55,56,57,58,59,60,61,62] have been outlined in Table 1. All studies were categorized as cross-sectional [22,40,41,42,43,44,46,47,48,49,50,51,52,53,54,55,56,57,58,59,60,61,62], with one of them utilizing a mixed methods approach (combining qualitative and quantitative data) [45]. The included studies were conducted on five out of seven continents, with four studies from the United States of America (USA) [43,55,61,62], two studies from Africa (South Africa and Kongo) [45,58], ten from Asia (China, India, Jordan, Nepal, Israel, Kingdom of Bahrain and Egypt, Palestine, Pakistan, Malaysia) [22,40,46,48,49,50,51,52,53,59] and eight from Europe (England, Italy, Greece, Belgium, Slovenia) [41,42,44,47,54,56,57,60]. Studies from Asia accounted for 42% of the studies analyzed (10 studies) [22,40,46,48,49,50,51,52,53,59]. The studies were published from January 2022 to August 2024.

### 3.3. Measures

Sociodemographic characteristics were recorded in all of the studies included [22,40,41,42,43,44,45,46,47,48,49,50,51,52,53,54,55,56,57,58,59,60,61,62]. The majority of the studies assessed the participants’ vaccination history, including the number of BDs received and the type of vaccine administered [22,40,46,47,48,52,53,57,58,59,60,61,62]. In addition, the studies included specific questions about willingness to take the COVID-19 vaccine BDs [22,40,48,49,51,52,54,57,58,59], vaccination trust [43,49,57], knowledge [50,53,54,57] and perceived benefit [22,46,47,49,53,56]. Questions related to attitudes about BDs [42,44,45,46,47,48,50,51,52,53,54,56,61], COVID-19 diagnosis and risk [44,46,60], barriers for BDs [46], health status factors [42,46,50,51,58,60] and psychological drivers for BDs [40] were also included and have been outlined in Table 1. Furthermore, specific assessment tools were used to evaluate vaccination hesitancy among HCPs, focusing on HCPs’ knowledge, attitudes, and other factors associated with it. In particular, the specific tools used were the Belief Medicine Questionnaire Specific (BMQ) and the Belief Medicine Questionnaire General (BMQ), the Brief Illness Perception Questionnaire (BIPQ), the Oxford COVID-19 Vaccine Hesitancy Scale, and the Bespoke scale [41].

### 3.4. Vaccination History and Hesitancy

Regarding vaccination history (Table 1), the majority of HCPs had received at least one dose of the initial COVID-19 vaccine [22,40,41,42,43,44,45,46,47,48,49,50,51,52,53,54,55,56,57,58,59,60,61,62]. The percentages of HCPs receiving booster vaccinations have been found to vary widely in studies, with reported rates ranging from 4.9% to 100%. More specifically, the first BD has been administered to a considerable percentage of HCPs [40,42,43,45,47,48,50,51,52,55,57,58,59,60,61,62], while a smaller proportion have received the second BD [40,41,46,47,50,54,57,58,62]. The number of HCPs who have received the third BD is even lower [41,46,57]. There were substantial variations in HCPs’ hesitancy towards BDs, ranging from 12% to 66.5% [22,40,42,44,45,49,50,51,52,53,54,56,57,58,59]. Hesitancy rates varied significantly across continents, with Asia ranging from 19.7% to 66.5%, Africa from 27% to 46.1%, and Europe from 14% to 60.2%. From the highest to the lowest rates of booster dose vaccination hesitancy, the countries were Palestine at 66.5% [22], Israel at 61.3% [51], Greece with rates ranging from 30.9 to 52.9% [44,56], Italy with rates ranging from 51.9% to 60.2%) [42,54], Egypt at 46.1% [53], Congo at 31.1% [58], Pakistan at 24.2% [40], South Africa at 20% [45], Malysia at 22% [59], South India at 19.7% [52], Jordan at 16% [49], Belgium at 14% [57], and Nepal at 12% [50]. Physicians displayed the most diverse hesitancy rates, ranging from 12.8% to 43.7%, whereas nurses and pharmacists exhibited rates between 26% and 37% and 26% to 34.6%, respectively.

### 3.5. Socio-Demographic Characteristics and COVID-19-Related Variables Associated with Vaccination Hesitancy

Socio-demographic characteristics related to vaccine booster dose hesitancy in HCPs were gender, age, type of HCPs included in studies, marital status, education level, co-morbidities, type of vaccine, and not being regularly vaccinated against influenza [22,40,42,43,44,45,46,47,49,50,51,52,53,54,55,56,57,58,59,60,62] (Table 2). When it comes to gender differences, females exhibited greater hesitancy towards receiving BDs than males [49,51,53,54,57,60]. Additionally, individuals without a chronic condition [33,45,58,60] and without medical training demonstrated a lower willingness compared to medical professionals to receive BDs [43,45,49,50,52,54,58]. Being in a younger age range [45,55,56,57,60,62], being single [40,52], and having a lower level of education [44,50,62] were also all factors that were positively associated with vaccine hesitancy. Family and friends were also identified as influential factors contributing to hesitancy [41,44,48,58]. On the other hand, individuals who had received mRNA-based vaccines in the past [40,46,47] and regularly received influenza vaccinations [22,44,45,55,56] were more inclined to be receptive to receiving a booster vaccine dose.

### 3.6. Vaccination Knowledge and Attitudes

In relation to vaccination attitudes, a notable percentage of HCPs (ranging from 6% to 77.1%) held the belief that the currently available BDs were not required, deemed unsafe, and lacked effectiveness [40,45,46,47,48,49,50,52,53,57,58] (Table 3). Furthermore, their belief was that vaccines do not provide adequate protection against severe cases of COVID-19 [40,42,51,52,57,59]. Consequently, there was a lack of trust in these vaccines, with percentages ranging from 20.9% to 26.8%. [43,44,49,57]. Fear of the side effects associated with COVID-19 booster vaccinations, [44,45,46,52,56], a belief in a low risk of COVID-19 infection [42,43,46,47,57], the non-compulsory nature of booster doses [41,43,44,52,56,57,59], and a history of COVID-19 infection [57] were also identified as contributing factors for vaccine hesitancy. Nevertheless, it seems that HCPs acknowledged receiving information about the efficacy of COVID-19 BDs [42], and expressed their willingness to receive additional information [42,51,54].

## 4. Discussion

The objective of this scoping review was to evaluate the hesitancy of HCPs towards vaccination with COVID-19 BDs and identify associated factors. Our findings suggest that HCPs exhibit varying degrees of hesitancy across countries, indicating that HCPs still had concerns related to BDs. This hesitancy leads to a progressive decrease in the percentage of HCPs receiving the second and third booster doses. The prevalence of hesitancy towards BDs in HCPs was higher among females, younger and single individuals, those with lower education levels, and those who did not regularly receive flu vaccines. Notably, individuals with a history of COVID-19 infection, without chronic conditions, and non-physician HCPs also exhibited hesitancy. This review also identified the following key factors that prominently influenced BD hesitancy: uncertainties surrounding the vaccine’s safety, efficacy, and necessity, a perception of low risk of contracting the infection, and that BDs were not mandatory.

In light of the evolving virus and the appearance of new variants, health authorities have endorsed the regular utilization of BDs to enhance and prolong vaccine-induced immunity [8,9,10]. However, our review indicates that a considerable portion of HCPs remain hesitant in receiving BDs of the COVID-19 vaccine. Literature research on vaccine hesitancy for COVID-19 in HCPs has produced varied results [63]. While HCPs generally exhibit lower vaccine hesitancy compared to non-healthcare workers [63], some studies have found no significant differences in vaccine hesitancy between these two groups [64,65]. Furthermore, the underlying factors contributing to vaccination hesitancy among HCPs appear to be similar to those documented within the general population [66]. This raises concerns since HCPs have traditionally been the primary and most trustworthy source of vaccine information [67]. It is to be expected that HCPs who have not been vaccinated are much less likely to suggest vaccinations to their patients [31]. However, even HCPs who have received their vaccinations need access to continually updated resources to effectively address vaccine hesitancy and discuss vaccines with their patients [31,68].

Another important finding of our review was the substantial variability in the hesitancy levels of HCPs across various countries. The COVID-19 vaccination hesitancy rates in Jordan at 16% [49], Belgium at 14% [57], and Nepal at 12% [50] were among the lowest worldwide, which could be attributed to significant efforts to build public trust in vaccines [69]. On the other hand, Palestine at 66.5% [22] and Israel at 61.3% [51] had the highest hesitancy rates. This variability aligns not only with other previous reviews conducted during the primary COVID-19 vaccination campaigns [12,70,71,72], but also with a subsequent review following the introduction of BDs [23]. Socioeconomic factors, such as race and income, are also significantly linked to geographic disparities in vaccine hesitancy [73]. A study analyzing COVID-19 vaccine hesitancy across 145 countries highlighted that hesitancy towards vaccination was a more prominent factor in determining uptake in low-income countries compared to high-income countries [74]. Lower availability or limited accessibility to COVID-19 vaccines [75,76], along with higher rates of COVID-19-related morbidity and mortality [70] in certain countries, may potentially explain the differences in hesitancy across countries regarding BD vaccinations. The widespread implementation of mandatory primary vaccinations for HCPs [77,78], and the resulting pressure to vaccinate, may also have contributed to hesitancy [77,79]. Nevertheless, and despite this considerable variation in hesitancy among HCPs worldwide, it is crucial to recognize the importance of implementing interventions that are tailored to each country’s socioeconomical [80,81,82] and even religious conditions [83].

A major finding of the present study was that HCPs continue to express concerns about the vaccine’s safety, necessity, and effectiveness, which are the same concerns that contributed to hesitancy towards the initial doses of the COVID-19 vaccine [12,70]. More specifically, HCPs have expressed concerns about negative effects of multiple boosters on the immune system [84,85], adverse events (AEs) and serious adverse events (SAEs) including myocarditis and pericarditis, particularly in younger males who have received mRNA vaccines [86,87]. There are also other rare but serious conditions, such as thrombosis with thrombocytopenia syndrome, that have contributed to unease among HCPs [88,89,90]. Although these AEs are statistically rare compared to the severe outcomes of COVID-19 infection (without a booster dose), they could have a significant impact on how HCPs perceive BDs [91,92,93,94,95]. To address these concerns and rebuild trust among HCPs, it is crucial to have transparent risk communication strategies and robust post-vaccine safety monitoring in place [96,97]. In support of this, evidence suggests that HCPs often express a desire for more convincing and comprehensive evidence, in terms of both quality and quantity, when deciding on vaccinations and whether to recommend them [42,98,99]. This emphasizes the necessity of ongoing training programs that focus on vaccine research, safety data, and effective communication strategies.

On the other hand, the belief that BDs of the COVID-19 vaccines are inadequate in providing protection against severe forms of COVID-19, as well as the lack of confidence in these vaccines, were also found to be significant factors contributing to vaccine hesitancy. Previous studies confirm these findings, since negative attitudes towards vaccines, lack of trust in government and institutions, and the belief that personal rights are being violated are all indicated as contributing factors to vaccine hesitancy [12,80]. Another important factor noted is the declining effectiveness of COVID-19 vaccines against infection as time goes on. Research has shown that vaccine-induced immunity, especially against new variants like Omicron, starts to decrease around five months following vaccination, after which “breakthrough” infections could occur [100]. Although breakthrough infections during this period are mostly mild, they have raised doubts about the long-term efficacy of BDs, especially for high-exposure groups like HCPs [100,101]. Behavioral science research indicates that deeper understanding has a stronger influence on decision making than statistical information, even among experts in the field [102]. This involves initiating a constructive dialogue, understanding the issues raised from the HCPs [103].

This review also found differences in vaccine hesitancy influenced by sociodemographic and medical history characteristics. More specifically, we found that characteristics such as female gender, lack of comorbidities, younger age, lower levels of education, being single, race/ethnicity (Blacks, Hispanics), and HCPs other than physician, have been identified as potential factors of vaccine hesitancy [25,26,27,28,29,30]. Consistent with prior research [40,45,46,47,50,51,53,54], female gender was identified as a key demographic factor contributing to hesitancy towards booster dose vaccination. In the past, women have shown more hesitancy towards receiving vaccinations for other diseases in comparison to men, and this tendency may also apply to the COVID-19 vaccine [104,105]. There are two main reasons for this. Firstly, the majority of reported side effects from the COVID-19 vaccine were observed in females, and secondly, women expressed concerns about the vaccine’s potential impact on fertility [105,106,107,108]. Importantly, having underlying health conditions also appears to be a factor in determining vaccine acceptance. In addition, it seemed that younger HCPs, who tended to have lower levels of education and no flu vaccination in the previous season showed a greater tendency towards being hesitant about BDs. On the other hand, there is a scarcity of studies that have focused on the contribution of different types of healthcare personnel to the reception of COVID-19 BDs. The findings of this review indicate that HCPs displayed varying levels of hesitancy towards COVID-19 vaccination, with non-physicians exhibiting higher levels of hesitancy compared to physicians.

Finally, it is important to consider that given the unique characteristics of the COVID-19 virus, achieving herd immunity through widespread vaccination presents significant challenges [109,110]. This is because COVID-19 has the ability to infect various animal reservoirs, including minks, deer, and rodents [111,112,113]. From this point of view, it becomes clear that the health of humans is intimately tied to the health of domestic, wild, and farmed animals [111,112,113]. Therefore, addressing COVID-19 requires a more holistic and internationally coordinated strategy, involving collaboration among diverse disciplines like medicine and veterinary medicine. This effort should be guided by the “One Health” concept, recognizing the inherent interconnectedness between human and animal health, and the ecosystem [114].

### Limitations

Our review makes a valuable contribution to the existing literature by thoroughly investigating the factors behind HCPs’ hesitancy towards COVID-19 booster dose vaccination. Nevertheless, it is essential to recognize certain limitations as well. To begin with, we exclusively examined articles published in English, thereby narrowing down the selection of eligible studies. Moreover, the studies we selected were drawn from different contexts and populations, which posed challenges in terms of making comparisons and conducting further analysis. The majority of studies have also used self-reported surveys, increasing the likelihood of response bias. Differences in reported vaccine hesitancy across countries could also be partly explained by variations in measurement techniques, including the use of different survey questions or assessment tools. Furthermore, as real-world data emerge, we should anticipate potential changes in HCPs’ views on COVID-19 vaccines. Longitudinal studies could offer valuable information about the evolving nature of attitudes in light of new developments and face-to-face interviews and focus groups could offer valuable insights into their beliefs and concerns that might not be captured by previous studies. Lastly, it should be noted that no evaluation was conducted on the articles’ quality, and the conclusions were simply summarized without any supplementary analysis.

## 5. Conclusions

In summary, our review underscores the hesitancy among healthcare professionals (HCPs) towards receiving booster doses of the COVID-19 vaccine despite receiving the initial dose of the COVID-19 vaccine. This hesitancy is primarily influenced by sociodemographic factors and concerns surrounding vaccine safety, necessity, and effectiveness. Gaining insight into these factors underlying this hesitancy could guide future vaccination approaches. To better understand the nuances of vaccine knowledge, attitudes, and behaviors, future research should adopt a longitudinal qualitative approach to examine variations across time and regions with new developments.

## Figures and Tables

**Table 1:** Characteristics of included studies (publication year, study type, country of origin, number of participants, vaccine history and hesitancy rates).

Author/Year (Ref.)	Study Type	Country	No of Participants	Vaccination History (%) Number of BDs Received	Vaccine Hesitancy (%)	Hesitancy Among HCPs by Specialty
Arshad et al., 2022 [40]	Quantitative	Pakistan	*n* = 1164	9.9% at least one BD	47.9%	24.4% Medical professionals 23.7% Allied Health Professionals
Dale et al., 2023 [41]	Quantitative	England	*n* = 91	77.1% at least one BD 4.9% 2 BDs 1.2% 3 BDs	Oxford Vaccine hesitancy scale score: 13.56/35.00 (17.1% being above the midpoint).	NR
Della Polla et al., 2022 [42]	Quantitative	Italy	*n* = 496	94.9% at least one BD 48.1% at least 2 BDs	47.4%	NR
Digregorio et al., 2024 [57]	Quantitative	Belgium	*n* = 1814	66.8% 2 BDs 6.3% 3 BDs	14%	NR
Dudley et al., 2023 [43]	Quantitative	USA	*n* = 1207	82% HCPs at least one BD	NR	6.3% Pediatrician 13% Family medicine 26.7% Physician assistant, Nurse Practitioners, and Nurse 26% Pharmacist
Galanis et al., 2023 [44]	Quantitative	Greece	*n* = 795	NR	30.9%	NR
George et al., 2023 [45]	Mixed-Methods	South Africa	*n* = 6235	56% at least one BD	27%	27.9% Nurse 17.5% Doctor
Gu et al., 2023 [46]	Quantitative	China	*n* = 1618	78.4% at least one BD	41.8%	43.7% Physician 37% Nurse
Guarducci et al., 2023 [47]	Quantitative	Italy	*n* = 1309	96.5% one BD	NR	NR
Kolomba et al., 2024 [58]	Quantitative	Congo	*n* = 514	24.3% one BD	31.1%	23.1% Doctor
Krishna et al., 2023 [48]	Quantitative	India	*n* = 535	62.2% one BD	40%	NR
Lubad et al., 2023 [49]	Quantitative	Jordan	*n* = 300	NR	31.6%	NR
Maraga et al., 2024 [22]	Quantitative	Palestine	*n* = 919	NR	66.5%	NR
Pandarathodiyil et al., 2024 [59]	Quantitative	Malaysa	*n* = 392	100% at least one BD	22%	NR
Paudel et al., 2023 [50]	Quantitative	Nepal	*n* = 300	29% one BD	12%	NR
Pristov al., 2024 [60]	Quantitative	Slovenia	*n* = 560	50.9% at least one BD	NR	NR
Ramot et al., 2023 [51]	Quantitative	Israel	*n* = 124	88.7% one BD 38.7% 2 BDs	61.3%	NR
Rathinakumar et al., 2024 [52]	Quantitative	South India	*n* = 572	12.6% one BD	19.7%	23.2% Paramedical workers 12.8% Doctor
Roberts et al. 2024 [61]	Quantitative	USA	*n* = 182	55% one BD	NR	NR
Russ et al., 2024 [62]	Quantitative	USA	*n* = 3375	85% one BD	NR	NR
Salah et al., 2023 [53]	Quantitative	Kingdom of Bahrain and Egypt	*n* = 389	NR	46.1%	46.1% Physicians 26% Nurses 34.6% Pharmacists
Sansone et al., 2024 [54]	Quantitative	Italy	*n* = 521	5.2% at least one BD	60.2%	NR
Viskupič et al., 2023 [55]	Quantitative	USA	*n* = 1084	63.2% one BD	NR	NR
Zoumpoulis et al., 2023 [56]	Quantitative	Greece	*n* = 1224	52.4% one BD 47.5% 2 BDs	27.4%	NR

BD: booster dose, HCP: healthcare professional, NR: not reported.

**Table 2:** Socio-demographic characteristics and COVID-19-related variables associated with vaccination hesitancy.

Factors	Associated with Hesitancy	Number of Studies
**Age**	Younger age [39,41,49,50,51,54,56].	7
**Gender**	Being male [34,39]. Being female [43,45,47,48,51,54].	8
**Race**	Black African [39]. Black [55]. Non-Hispanic Black [56]. Hispanic [56].	3
**Education**	Lower education [38,44,56].	3
**Occupation**	Non-prescribers [51]. Other than physicians [37,38,39,43,44,46,48,52]. Physicians [41,45,47]. Pharmacists [47]. Νot in direct contact with patients [39]. Less job experience [39]. Working experience more than 5 years [52]. Wards of activity with lower risk of infection (Medical vs. Emergency/Critical/Infectious Disease wards) [48].	12
**Political Leanings**	Republican self-identification [49].	1
**Marital Status**	Single/not married [34,46]. Married [38].	3
**Friends/family**	Influence of friends/family [35,38,42,52].	4
**Area of Residence**	Rural [34,37]. Highly socially vulnerable census tract [56].	3
**Low income**	Low annual household income (<USD 50,000) [56].	1
**Comorbidity/chronic illness**	Absence of chronic conditions [38,39,52,54]. Permanent or temporary medical conditions [37]. History of allergy [40]. Obesity [47].	7
**Health status**	Good/very good self-perceived physical health [38]. Unhealthy dietary habits [54].	2
**Time constraints**	Lack of time [35]	1
**Flu Vaccination**	Lack of flu vaccination [17,38,39,49,50].	5
**Hygiene measures**	Increased compliance [38,41,52].	3
**Relating to COVID-19**	Previous COVID-19 infection [34,35,49]. No previous infection [38].	4
**Relating to COVID-19 vaccination**	No previous vaccination [34] Type of vaccine (non-mRNA COVID-19 vaccines) [34,53] Less previous vaccine doses [44,48,51,52] Uptake of the first booster dose [42] Previous side effects [34]	10

**Table 3:** Knowledge/attitudes associated with vaccine hesitancy.

Knowledge/Attitudes	Associated with Hesitancy	Number of Studies
**Trust-related issues**	Vaccine safety [34,35,38,39,40,41,42,43,45,47,52].	11
	Pregnancy safety [35].	
	Vaccine effectiveness [17,34,36,38,39,40,41,42,43,45,46,47,51,52,53,55].	16
	Vaccine necessity [35,36,38,39,40,45,46,47,52,55].	10
	Vaccine Side effects [17,36,37,46,50,52,55].	7
	Mistrust in government/scientists [37,42,43,50].	4
	Rapid development of the vaccines [37,42,50].	3
	Distrust due to racism and previous unethical treatment of minorities [37].	1
	Reliability of clinical trials (not including HCPs) [37].	1
	Low trust and satisfaction in COVID-19 vaccination [34,38,51].	3
	Wanting to wait more [37,42,43,45].	4
**Information**	Lack of information/misinformation [42,47,48].	3
**Beliefs and attitudes about health and prevention**	Low risk of COVID-19 infection [36,37,40,50,51].	5
	Immune system capable of fighting COVID-19 [43,51].	1
	Lower perception of the severity of COVID-19 [36,41,51,52].	4
	Against vaccines in general [50,52].	2
	Belief in greater efficacy of complementary alternative medicine [50].	1
**Ethics**	Mandatory Vaccination [39,41,48].	3
**Other**	Not being very likely to suggest the vaccine to patients [51].	1
	Tiredness due to the vaccination procedure [38].	1

## Supplementary Materials

The following supporting information can be downloaded at: https://www.mdpi.com/article/10.3390/vaccines12121411/s1, Table S1: Preferred Reporting Items for Systematic reviews and Meta-Analyses extension for Scoping Reviews (PRISMA-ScR) Checklist.
